# A Rare Variant of Caval Malformation: Left-Dominant Caval System With Anomalous Cardiac Drainage

**DOI:** 10.7759/cureus.89973

**Published:** 2025-08-13

**Authors:** Garrison Allen, Pranav Shah, Rajiv Biswal

**Affiliations:** 1 Radiology, Hackensack Meridian School of Medicine, Hackensack, USA; 2 Radiology, Jersey Shore University Medical Center, Neptune, USA

**Keywords:** congenital vascular anomaly, coronary sinus (cs), hemiazygos continuation, left-sided inferior vena cava, persistent left-sided superior vena cava (plsvc)

## Abstract

In aggregate, caval malformations are relatively common congenital abnormalities that exist on a spectrum of severity and rarity. These result from inappropriate persistence or regression of bilateral primitive caval structures. Here, we present the case of a 44-year-old woman with a complex cardiovascular medical history who presented for a fall with bilateral subdural hematomas. During workup, she was found to have an extremely rare variant of caval malformation, including both left-sided inferior vena cava (IVC) with hemiazygos continuation and left-sided superior vena cava (SVC). This case serves as an opportunity to learn about the embryology and pathology associated with caval malformations. Ultimately, these anomalies were determined not to be implicated in her fall or the treatment of subdural hematomas but raise questions when considering the treatment of her cardiovascular comorbidities.

## Introduction

The inferior vena cava (IVC) is the primary venous structure that drains the lower extremities and viscera. It typically meets up with its counterpart, the superior vena cava (SVC), at the cavoatrial junction and additionally communicates through the azygos and hemiazygos veins. Both the IVC and SVC are normally right-sided structures. Congenital anomalies in central veins are relatively common anatomic abnormalities with an estimated prevalence of up to 10% [1]. However, caval abnormalities encompass a plethora of different vascular anomalies that can broadly be divided into left-sided, duplicate, or discontinuous systems, with some being more common than others. On their own, these variations are usually benign but may complicate procedures that involve central venous access or be associated with medical conditions such as congenital heart disease. Left-sided IVC is one of these anomalies that occurs in approximately 0.2%-0.5% of individuals [2]. Hemiazygos continuation of the IVC may also occur, in which the IVC effectively ends at the level of the renal veins and connects into the hemiazygos vein [3,4]. This appears as an IVC that ascends completely on the left side through both the abdomen and thorax. Similarly, left-sided SVC occurs in approximately 0.5% of individuals and up to 10% in those with congenital heart defects [5]. Rarely are these anomalies observed together outside of situs inversus, where the sidedness of major structures and organ systems is reversed. Here, we present a case of a left-sided IVC with hemiazygos continuation and corresponding left-sided SVC. On literature review, only one similar case was found to have been described previously [6]. However, in this example, the left SVC drains through an unknown tract directly into the right atrium, as compared to the vast majority of left-sided SVC cases, which drain through the coronary sinus.

## Case presentation

**Figure 1:**
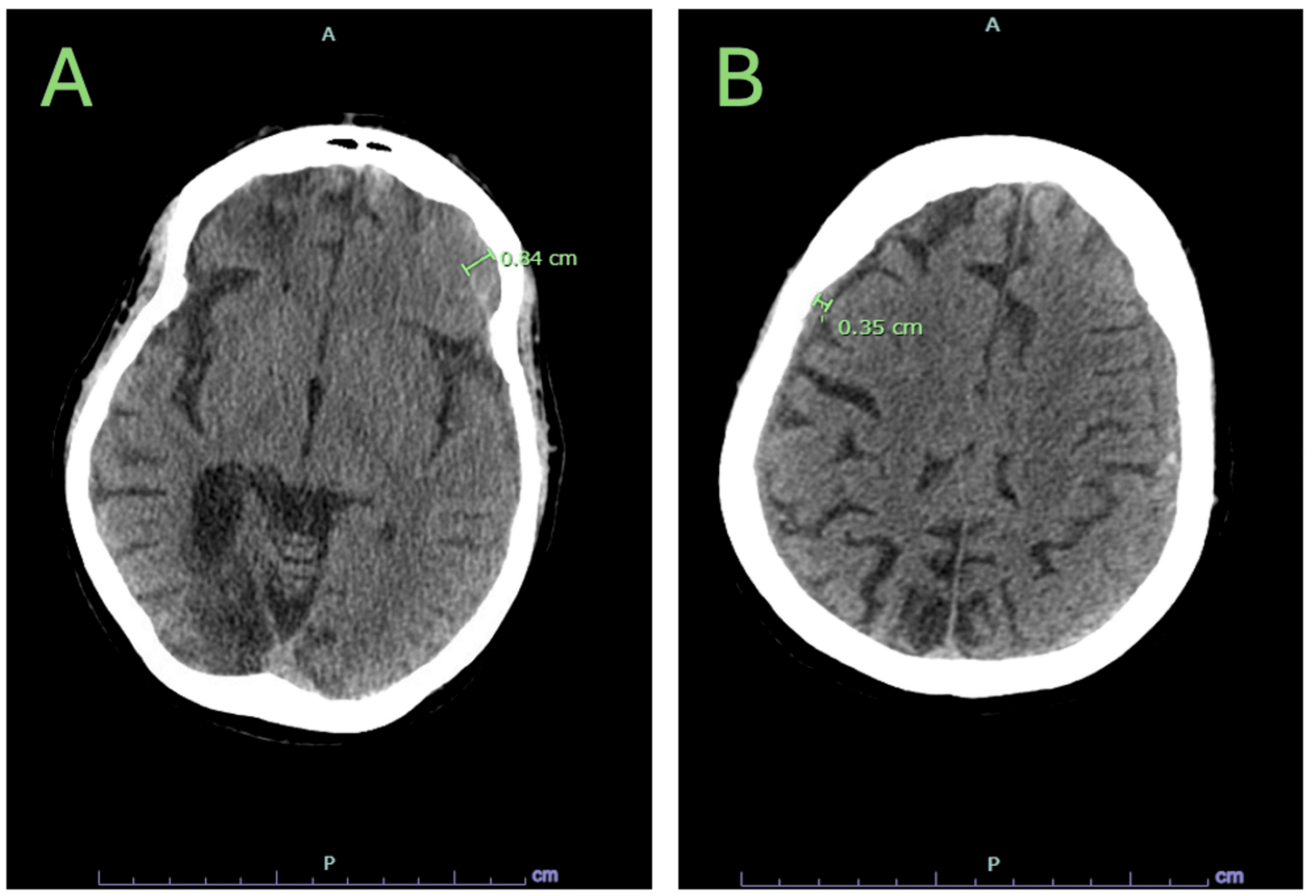
Subdural hematomas demonstrated on the left (A) and right (B).

In this case, a 44-year-old woman presented to the emergency department (ED) for a fall from standing, with trauma to her head and face. She has a complicated past medical history, including a congenital heart defect with congestive heart failure, chronic atrial fibrillation (AFib) on apixaban, and seizure disorder. Surgical history was notable for patent foramen ovale closure and pacemaker placement. Due to her medical comorbidities, she resides at an assisted living facility and uses a walker, but is independent in all activities of daily living. On arrival to the ED, she was found to have suffered a head strike resulting in nasal bridge laceration and periorbital ecchymosis, but was alert and oriented with an intact memory of the event and no neurological deficits from baseline. She denied loss of consciousness. Vitals on arrival were stable, with blood pressure (BP) of 95/53, pulse of 74, temperature of 98.2°F, respiratory rate of 20, and oxygen saturation of 96%. Laboratory results were unremarkable, with no anemia, thrombocytopenia, or electrolyte abnormalities. X-ray imaging of the right knee showed no fractures, and computed tomography (CT) of the facial bones demonstrated a non-displaced fracture of the right nasal bone. Despite a lack of neurological impairment, CT of the head without contrast revealed mild bilateral subdural hematomas (Figure 1). Apixaban was subsequently reversed with recombinant factor Xa, and she was transferred to a level 1 trauma center for a higher level of care.

**Figure 2:**
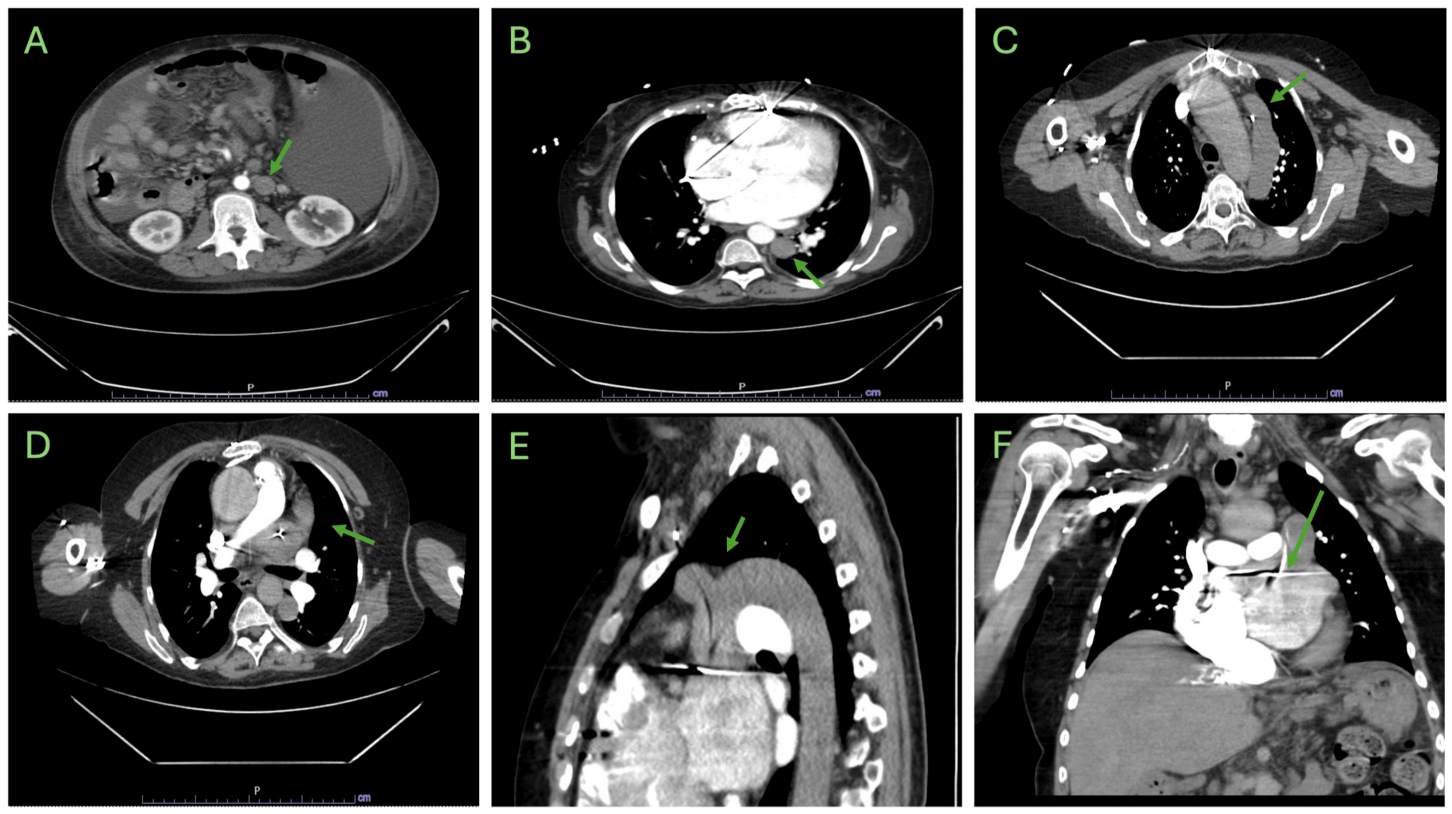
IVC is seen located slightly left and posterior to the aorta on contrast CT (A), continuing into the thorax along the hemiazygos course (B). Left-sided SVC is seen adjacent to the aorta (C), meeting up with the IVC and coursing directly into the right atrium (D). The IVC is seen connecting with the left SVC through an azygos arch (E) and draining into the right atrium through an unknown tract, highlighted by the path of the pacemaker lead (F). IVC: inferior vena cava, CT: computed tomography, SVC: superior vena cava

While at the trauma center, her condition was stable, and she underwent additional workup for abdominal ascites of unknown origin. Subsequent imaging demonstrated multiple venous anomalies, including an anteriorly positioned hepatic portal vein and, most notably, a left-sided IVC with hemiazygos continuation (Figure 2). Imaging during a later admission revealed a left-sided SVC that joins with the previously seen hemiazygos IVC before crossing directly into the right atrium.

During her hospital stay, she remained stable with no complications and was discharged to subacute rehabilitation on day 6. Since then, she has had multiple hospital readmissions for musculoskeletal complaints and seizures related to her underlying seizure disorder. No further imaging has been performed to identify the tract.

## Discussion

The caval system is derived from a system of primitive venous structures known as the cardinal, vitelline, and umbilical veins [7]. These systems begin as large as symmetric structures with regression of left-sided segments, leaving the right-dominant venous system. Abnormal regression or persistence of one or multiple of the primitive venous structures will result in a congenital venous anomaly, which broadly includes left-sided, duplicate, or discontinuous venous systems. In this case, we describe an occurrence of caval anomaly that includes left-sided IVC with hemiazygos continuation in conjunction with a persistent left SVC. In approximately 90% of cases, a left-sided SVC drains into the right atrium through the coronary sinus. The majority of the remaining 10% drains directly into the left atrium, creating a right-to-left shunt. For duplicate SVC, drainage patterns are variable but commonly drain through these same routes and may have communication through the left brachiocephalic vein [8]. In this case, the left IVC and SVC join in an azygous arch structure, which then drains into the right atrium via an unknown tract, highlighted by the placement of pacing leads. Given the anterior position of this structure, it is unlikely to be the coronary sinus. However, further imaging would need to be performed to definitively identify this tract.

While congenital caval abnormalities are most often asymptomatic and discovered as incidental findings on imaging, some are associated with diseases of venous insufficiency or thrombosis [9]. In one case, left-sided IVC was implicated as the cause of nutcracker syndrome in an 11-year-old boy due to compression of the IVC between the superior mesenteric artery and aorta [10]. Beyond possible implications of functional venous disorders, central venous anomalies pose a significant challenge for intravascular procedures. If the unusual anatomy is not recognized prior to intervention, the patient may be subjected to unnecessary harm. For instance, in the placement of an internal jugular catheter, the device is typically passed from the right internal jugular vein into the SVC and positioned at the cavoatrial junction with confirmation of appropriate placement by chest X-ray. In addition, placement of an IVC filter requires a pre-placement IVC evaluation to identify patency and configuration of the IVC, as well as to identify concurrent anomalies such as retro-aortic or circum-aortic renal veins for proper positioning of the filter. Failure of routine device placement due to anatomic abnormalities may result in, at the very least, an unnecessary procedure or even perforation of a great vessel. While complications such as perforation of a great vessel are rare, the high mortality risk necessitates consideration [11]. Similar risks may also exist with other intravascular procedures, although early detailed imaging in those cases could elucidate the unusual anatomy.

## Conclusions

This case demonstrates a rare constellation of caval malformations: left-sided IVC with hemiazygos continuation into persistent left SVC with anomalous cardiac drainage. While these congenital venous malformations were incidental and likely unrelated to the fall and subdural hematomas that the patient presented with, this case demonstrates the importance of recognizing vascular variants for clinical planning. Most notably with intravascular procedures such as pacemaker or IVC filter placement, these anomalies complicate the procedure. Successful identification is necessary to guide safe and effective care.
